# Supplementary material for: Synthesis and Biological Evaluation of 2,3,4-Triaryl-1,2,4-oxadiazol-5-ones as p38 MAPK Inhibitors
Source: Molecules. 2021 Mar 20;26(6):1745. doi: 10.3390/molecules26061745 (PMC8003627; doi:10.3390/molecules26061745)
Supplement: Supplementary file 1 [file molecules-26-01745-s001.pdf]

Electronic Supplementary Material

to

Synthesis and Biological Evaluation of 2,3,4-triaryl-1,2,4-oxadiazol-5-ones as p38 MAPK inhibitors

Roberto Romeo [1,*], Salvatore V. Giofrè [1], Maria A. Chiacchio [2], Lucia Veltri [3], Consuelo Celesti [4] and Daniela Iannazzo [4]

[1] Dipartimento di Scienze Chimiche, Biologiche, Farmaceutiche ed Ambientali, Università di Messina, Via S.S. Annunziata, 98168 Messina, Italy; robromeo@unime.it (R.R.); sgiofre@unime.it (S.V.G.); robromeo@unime.it (L. L.)

[2] Dipartimento Scienze del Farmaco, Università di Catania, Viale A. Doria 6, 95125 Catania, Italy; ma.chiacchio@unict.it (M. A. C.)

[3] Dipartimento di Chimica e Tecnologie Chimiche, Università della Calabria, Via P. Bucci 12/C, 87036 Arcavacata di Rende, Italy; lucia.veltri@unical.it (L. V.)

[4] Dipartimento di Ingegneria, Università di Messina, Contrada Di Dio, 98166 Messina, Italy; ccelesti@unime.it (C. C.); diannazzo@unime.it (D. I.)

* Correspondence: robromeo@unime.it (R. R.); Tel.: +39-090-676-6565

Table of Contents

^{1}H NMR spectra of compounds **3c-l**……………………………………………………………S2-S5

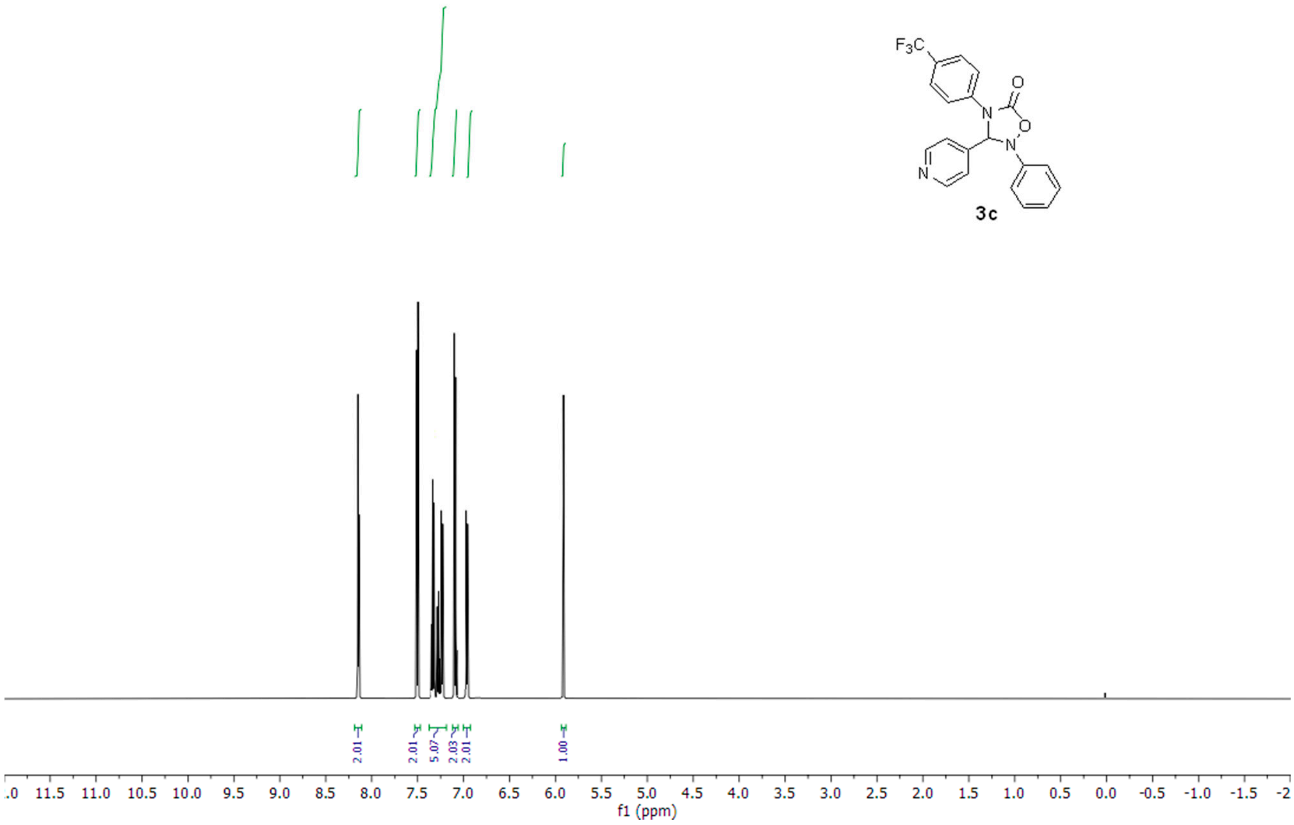

Figure S1. ^{1}H NMR spectrum of **3c** in $CDCl_3$, recorded at 25°C and 500 MHz

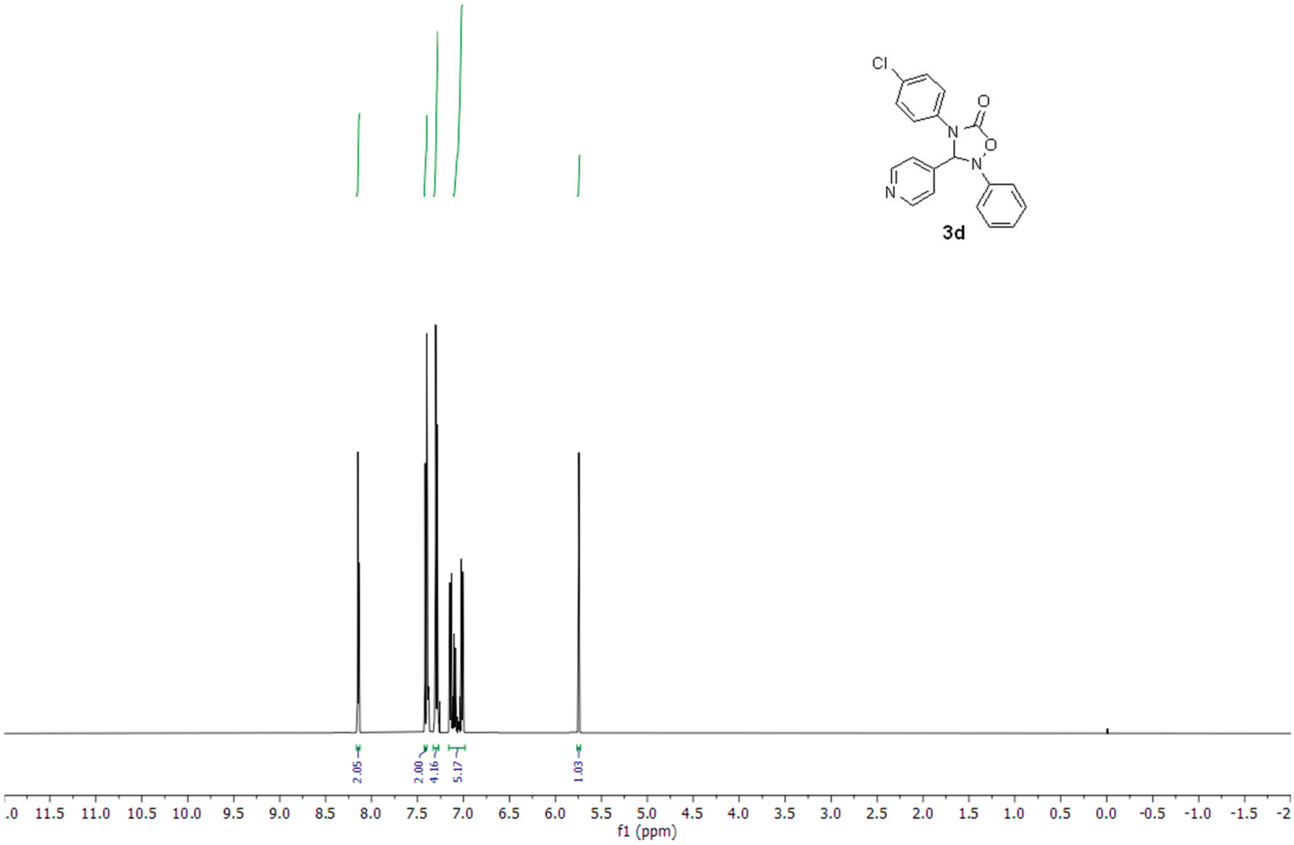

Figure S2. ^{1}H NMR spectrum of **3d** in $CDCl_3$, recorded at 25°C and 500 MHz

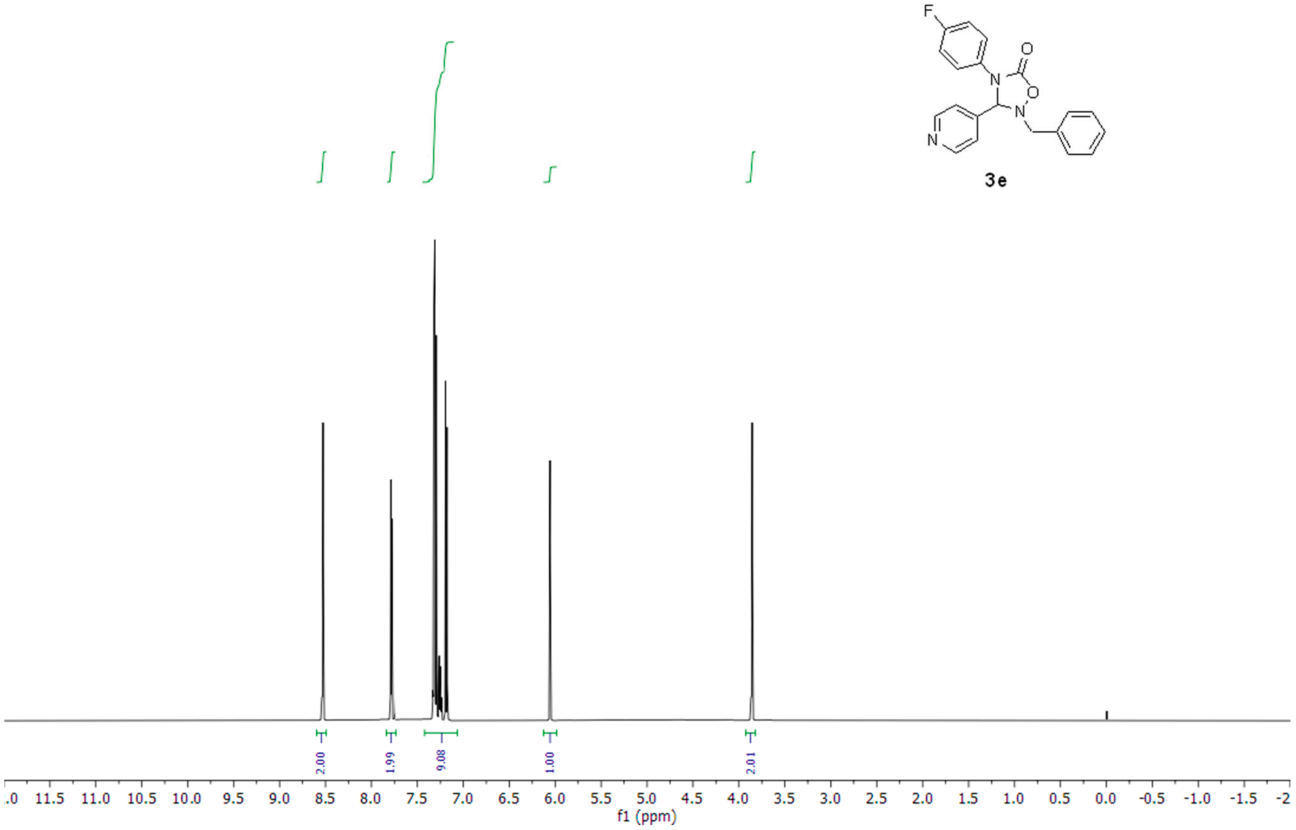

Figure S3. ^{1}H NMR spectrum of **3e** in $CDCl_3$, recorded at 25°C and 500 MHz

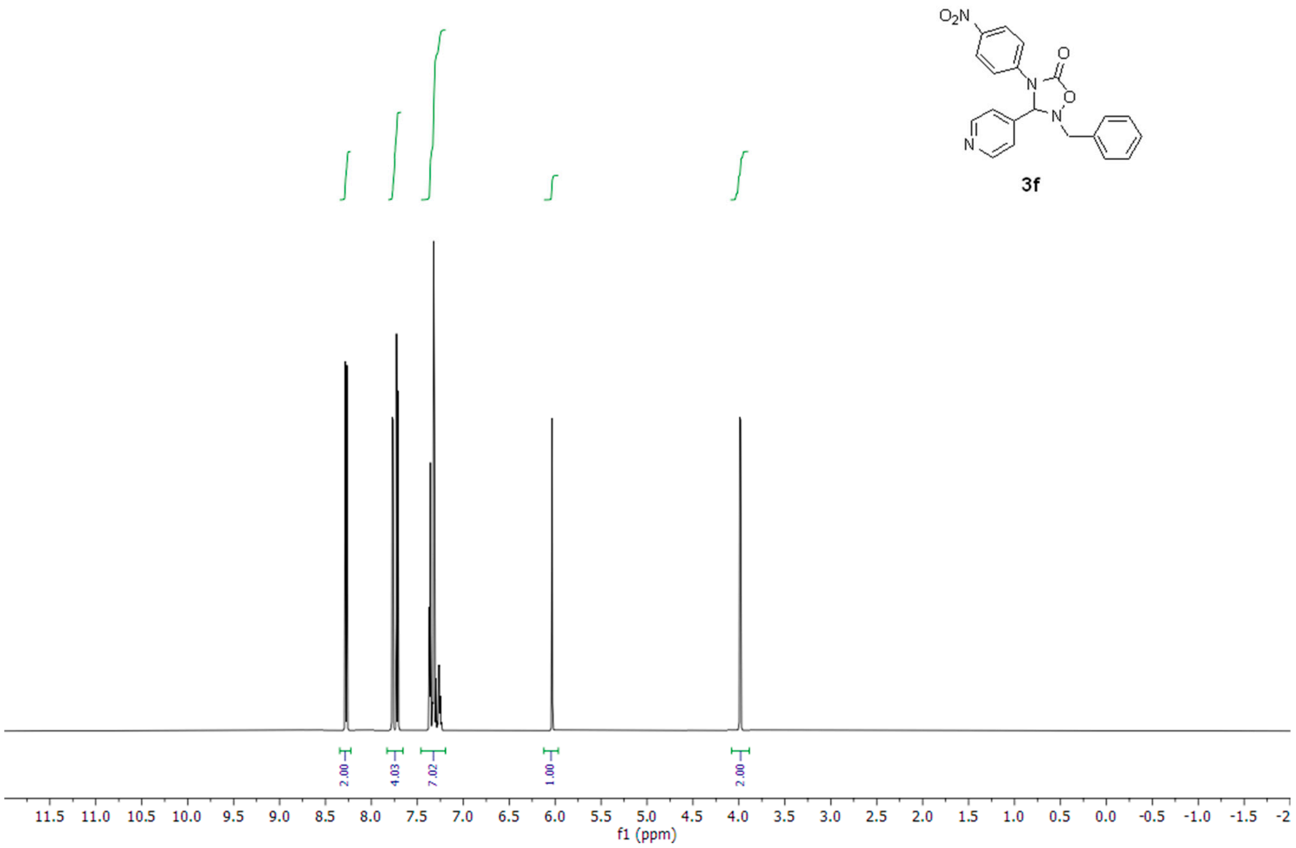

Figure S4. ^{1}H NMR spectrum of **3f** in $CDCl_3$, recorded at 25°C and 500 MHz

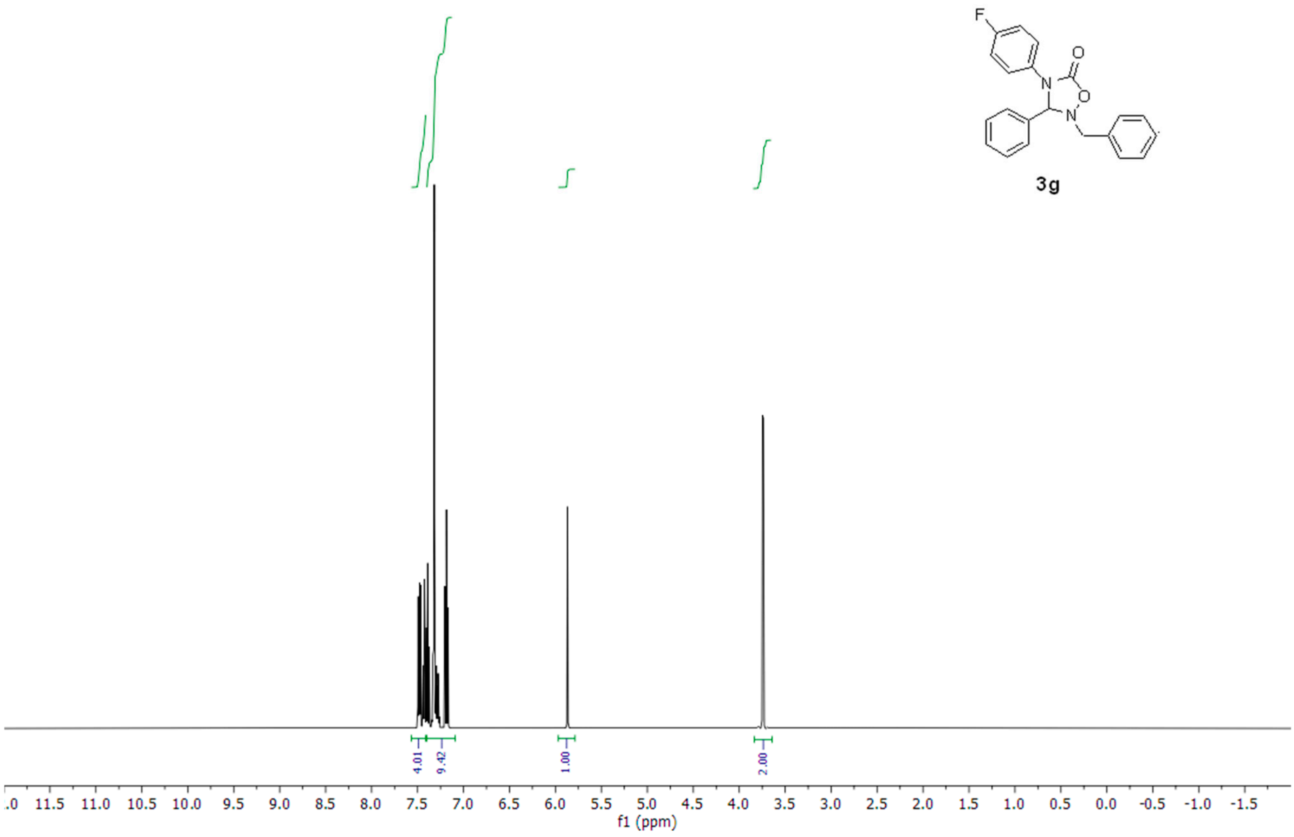

Figure S5. ^{1}H NMR spectrum of **3g** in $CDCl_3$, recorded at 25°C and 500 MHz

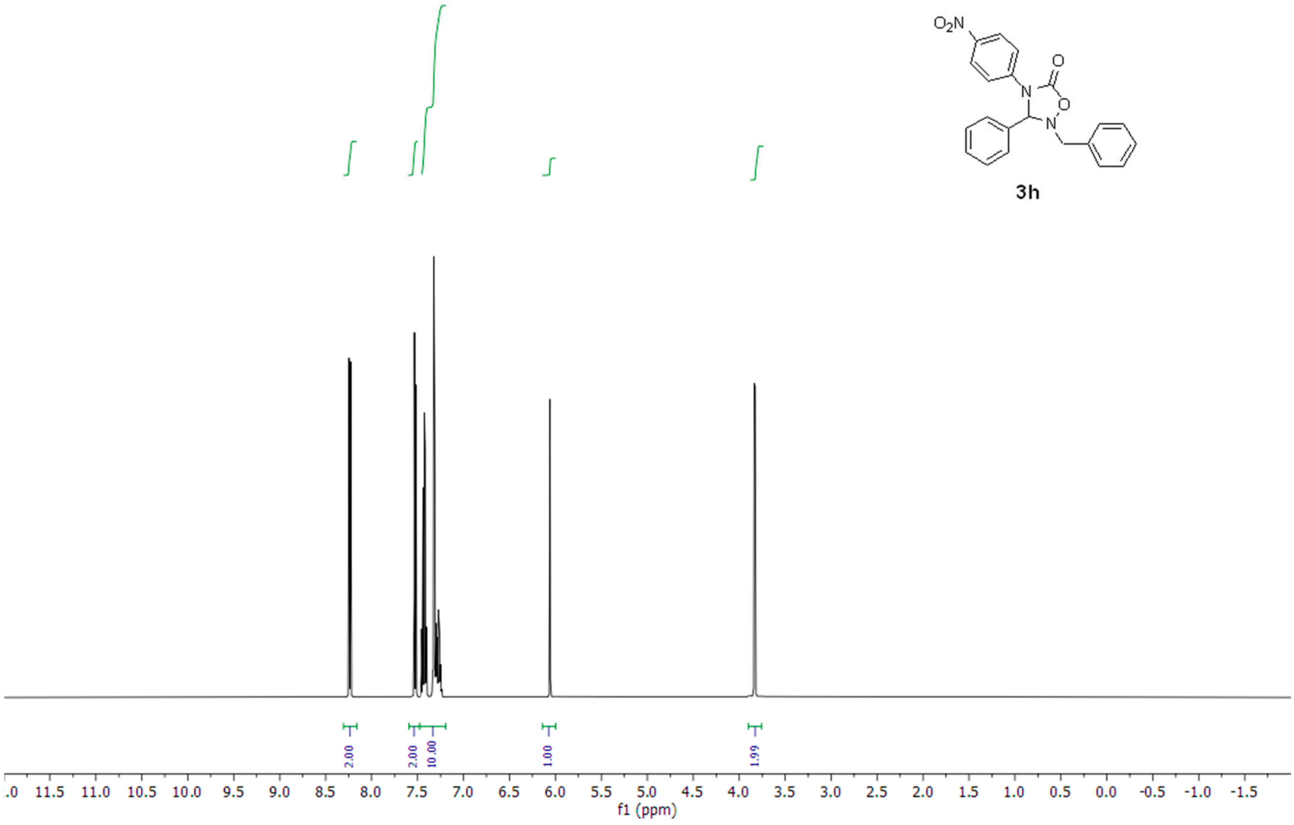

Figure S6. ^{1}H NMR spectrum of **3h** in $CDCl_3$, recorded at 25°C and 500 MHz

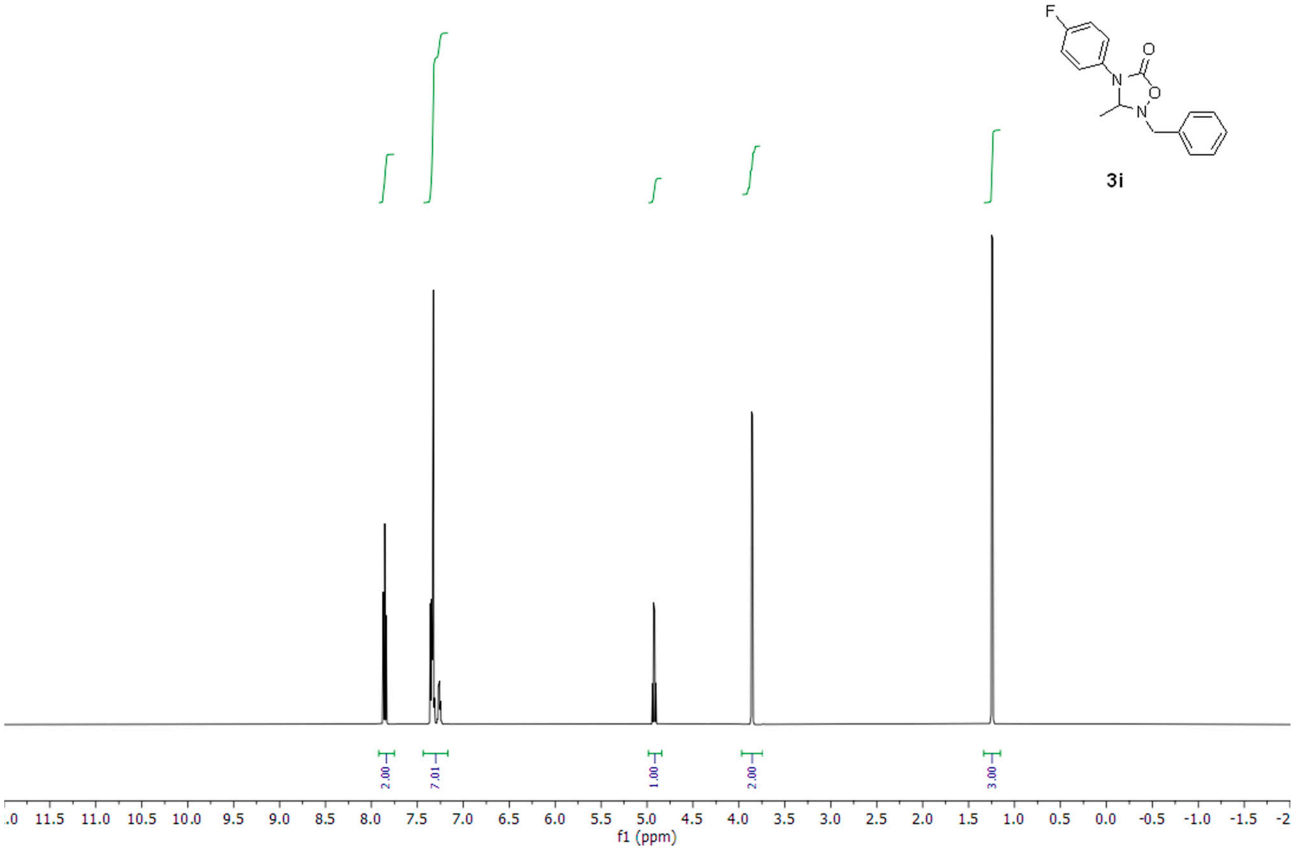

Figure S7. ^{1}H NMR spectrum of **3i** in $CDCl_3$, recorded at 25°C and 500 MHz

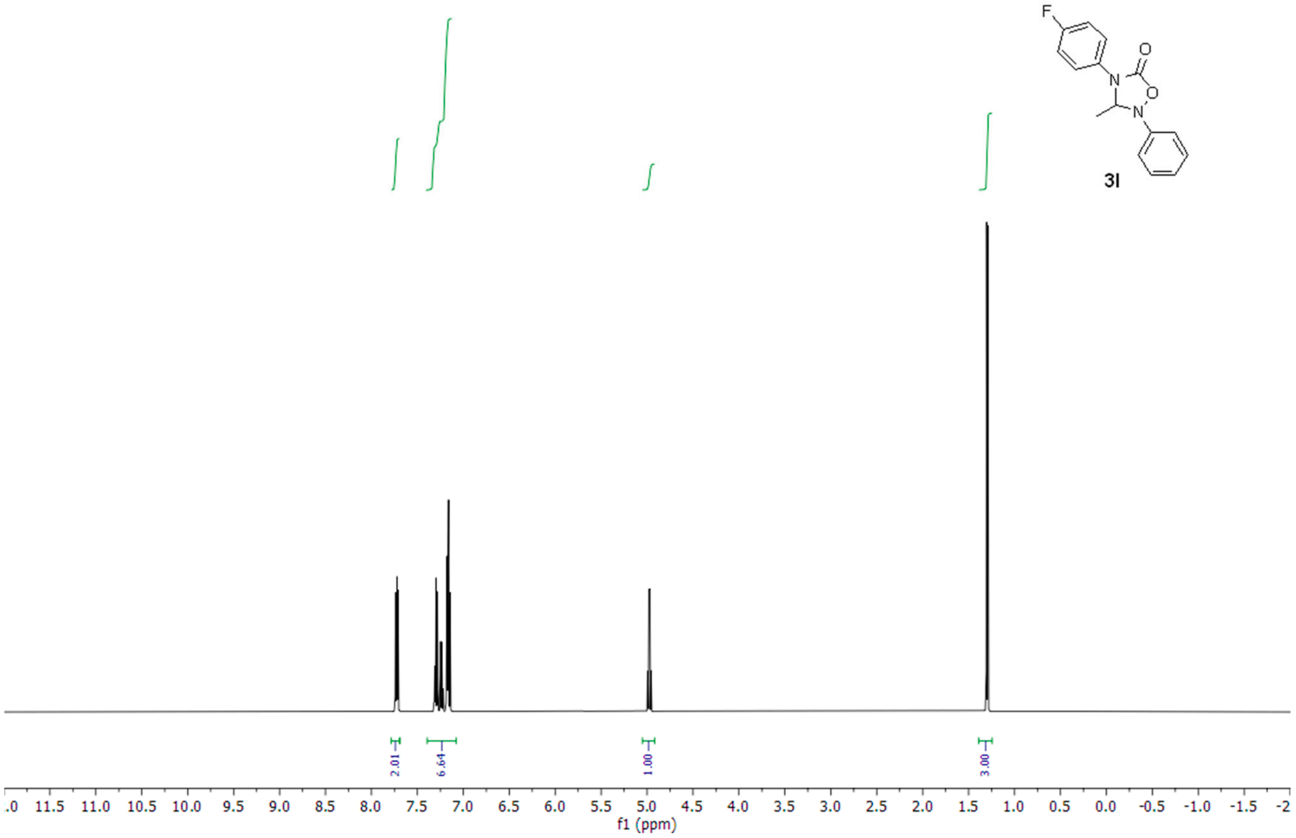

Figure S8. ^{1}H NMR spectrum of **3l** in $CDCl_3$, recorded at 25°C and 500 MHz
